# An isoform-specific allele of the *sax-7* locus

**DOI:** 10.17912/micropub.biology.000092

**Published:** 2019-03-27

**Authors:** Dylan Rahe, Ines Carrera, Filip Cosmanescu, Oliver Hobert

**Affiliations:** 1 Department of Biological Sciences, Columbia University, New York, NY, USA, Howard Hughes Medical Institute; 2 Department of Biochemistry and Molecular Biophysics, Columbia University Medical Center, New York, NY, USA; 3 Current Address: School of Chemistry, University of the Republic, Montevideo, Uruguay

**Figure 1:**
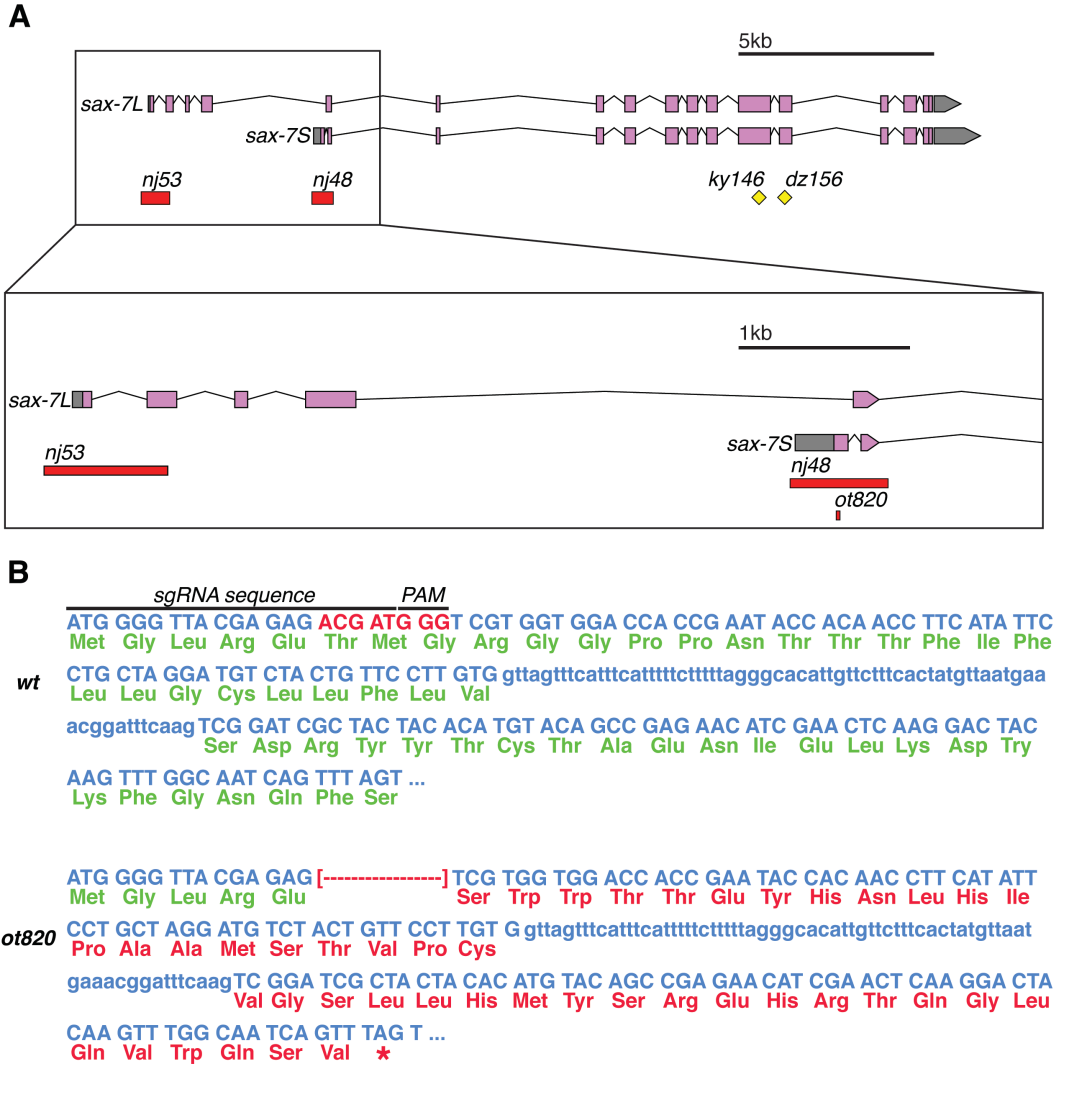
***ot820*** **is a novel isoform-specific allele of the *sax-7* locus.** **A.** *sax-7* locus encodes two isoforms of an L1CAM homolog. Existing null alleles affect either the long isoform (*nj53*) or both isoforms (*nj48, ky146,* and *dz156*). *ot820* is a short deletion mutant that affects only the short isoform. **B.** sgRNA targets the beginning of the first exon of *sax-7S*, and *ot820* is a 8bp deletion 15bp from the start codon.

## Description

The immunoglobulin superfamily member *sax-7* produces a long and short isoform that appear to have distinct functions (Chen *et al.* 2001; Wang *et al.* 2005; Sasakura *et al.* 2005; Pocock *et al.* 2008). An isoform-specific allele for the long isoform, *sax-7L*, was previously reported (Sasakura *et al.* 2005), but an isoform-specific allele for the short isoform, *sax-7S*, has been lacking (Fig. 1A). We used CRISPR/Cas9 to generate such an allele, using the *unc-22* co-CRISPR method (Kim *et al.* 2014). The *ot820* allele was isolated using sgRNA targeted to the first exon of *sax-7S* (sgRNA sequece: 5’ – TGGGGTTACGAGAGACGAT – 3’). Twitching progeny were screened by PCR and Sanger sequencing, and an 8bp deletion 15bp from the start codon was isolated (screening primers: 5’ – GGTGCTTCTCTGGTGGTAGC – 3’ and 5’ – TGTTGGCAAACAAAATACACG – 3’, Fig. 1B). While the *sax-7L* isoform is predicted to be entirely unaffected by this allele, the resultant frameshift is predicted to generate a 49 amino acid protein in which all but the first five amino acids of *sax-7S* are aberrant and has no predicted signal sequence, before terminating in a premature stop in the second exon (Fig. 1B). This allele therefore likely represents a null for the *sax-7S* isoform. After we generated this allele, a recent paper reported an additional *sax-7S*-specific allele, with somewhat similar, but not identical sequence properties (Chen et al., 2019). Consistent with previous evidence of distinct functions of *sax-7* isoforms, these authors showed differing axon fasciculation defects between these two alleles, which is further distinct from a total null allele. These new *sax-7S* alleles should help to reveal new insights into the role of L1CAM/SAX-7 isoforms in the nervous system.

## Reagents

OH13830 *sax-7(ot820) IV; oyIs14 V*. Will be available at CGC.
